# The Influence of Acute Oral Lactate Supplementation on Responses to Cycle Ergometer Exercise: A Randomized, Crossover Pilot Clinical Trial

**DOI:** 10.3390/nu16162624

**Published:** 2024-08-09

**Authors:** Taylor R. Ewell, Matthew C. Bomar, David M. Brown, Reagan L. Brown, Beatrice S. Kwarteng, David P. Thomson, Christopher Bell

**Affiliations:** Department of Health and Exercise Science, Colorado State University, Fort Collins, CO 80523-1582, USA; taylor.ewell@colostate.edu (T.R.E.);

**Keywords:** supplement, ergogenic, ergolytic, VO_2peak_, performance, FTP^20^

## Abstract

The purpose of this study was to investigate the potential ergogenic effects of an oral lactate supplement. For this double-blind, randomized, placebo-controlled crossover design, fifteen recreational exercisers (nine males, six females) ingested a placebo or a commercially available lactate supplement prior to cycle ergometer exercise. Primary outcomes included peak oxygen uptake (VO_2peak_; via indirect calorimetry), VO_2_ at the ventilatory threshold, and work rate at the lactate threshold (arterialized venous blood from a heated hand) determined during incremental exercise to fatigue, and power output during a 20-min cycling time trial. Compared with placebo, the oral lactate supplement (19 ± 1 mg/kg body mass) did not influence VO_2peak_ (placebo: 44.3 ± 7.8 vs. oral lactate: 44.3 ± 7.1 mL/kg/min (mean ± SD); *p* = 0.87), VO_2_ at the ventilatory threshold (placebo: 1.63 ± 0.25 vs. oral lactate: 1.65 ± 0.23 L/min; *p* = 0.82), or work rate at the lactate threshold (placebo: 179 ± 69 vs. oral lactate: 179 ± 59 W; *p* = 0.41). Throughout the 20-min time trial, the work rate was slightly greater (4%) with oral lactate (204 ± 41 W) compared with placebo (197 ± 41 W; main effect of treatment *p* = 0.02). Collectively, these data suggest that this commercially available lactate supplement did not acutely influence the physiological responses to incremental cycle ergometer exercise but elicited a modest ergogenic effect during the short-duration time trial.

## 1. Introduction

Lactate has been incorrectly characterized as a facilitator of fatigue and harbinger of muscle soreness. In contemporary physiology, the appreciation of lactate has evolved, and it is now recognized, amongst other things, as a versatile fuel source, a signaling molecule [1], a potential regulator of clinically relevant myokines such as interleukin-6 [2,3] and fibroblast growth factor-21 [4], and a promoter of buffering capacity [5,6]. Considering these favorable and physiologically relevant properties, lactate supplementation has been proposed as a potential ergogenic aid [5,7,8,9] and, based on observations in rodents, as an intervention to promote adaptation to exercise training [10,11]. To date, the human exercise data are somewhat limited. Several studies have explored physiological responses to lactate infusions, but these have typically focused on clinical and/or metabolic outcomes rather than exercise performance [12,13,14,15,16]. While a moderate number of studies have explored oral lactate administration [5,6,8,9,17,18,19], the results have been inconclusive. Further, none have examined the influence of oral lactate on the cardiometabolic responses to incremental exercise, such as peak oxygen uptake (VO_2peak_) or lactate and ventilatory thresholds.

The purpose of this study was to investigate the potential ergogenic effects of a commercially available oral lactate supplement in healthy young adults who were regular exercisers. The primary outcomes were conventional parameters that have been established as predictors of endurance performance and markers of training status: VO_2peak_, VO_2_ at ventilatory threshold, work rate at lactate threshold, and the maximal mean power that could be sustained during a 20-min stationary cycle ergometer time trial. We hypothesized that acute oral lactate supplementation would favorably modify each of these outcomes relative to a placebo.

## 2. Materials and Methods

### 2.1. Participants

This project was approved by the Institutional Review Board of Colorado State University (Protocol #4933H), conducted according to the guidelines of the Declaration of Helsinki, and registered as a clinical trial (NCT06371521). Written informed consent was provided by all participants prior to the initiation of any research activities. Healthy young adults were prospectively recruited by T.R.E., M.C.B., D.M.B., R.L.B., and B.S.K. Inclusion criteria consisted of age between 18 and 30 years old, and participation in a minimum of 150 min/week of moderate-intensity endurance exercise during the previous twelve months. Exclusion criteria included pregnancy, breastfeeding, use of an oral lactate supplement within the previous 4 weeks, use of magnesium supplements within the previous 4 weeks, unwillingness to abstain from the use of other potentially ergogenic supplements throughout participation in the study, and inability or unwillingness to perform vigorous cycle ergometer exercise.

### 2.2. Brief Summary of Protocol and Experimental Design

A randomized, double-blind, placebo-controlled, crossover design was used to minimize the influence of between-participant variation typically associated with a parallel design [20]. Further, although crossover designs are often associated with a relatively larger participant dropout [20], this was not anticipated in the current study on account of the brief nature of the intervention. All data were collected in the laboratories of the Department of Health and Exercise Science at Colorado State University, Fort Collins, CO, USA. Following screening, study participants were habituated to two stationary cycle ergometer exercise protocols: a ramp test and a 20-min time trial. Over four separate visits, participants then completed two incremental exercise tests for voluntary fatigue and two endurance performance tests requiring a maximal sustained effort over 20 min (similar to a 20-min functional threshold power test (FTP^20^)). Thirty minutes prior to each test, participants ingested capsules containing either a placebo or a commercially available lactate supplement (Sportlegs, Sport Specifics Inc., Longmont, CO, USA). To avoid the potential confounding influence of diurnal fluctuations, the scheduling of the incremental exercise tests and time trials was kept constant (within ~2 h) for each individual participant. The washout period (i.e., the time between trials) was a minimum of 48 h; across participants, it ranged from 48 h to 7 days. The methods were not changed after trial commencement. 

### 2.3. Randomization

The assignment order of placebo and oral lactate was randomized by T.R.E., using an online randomizer (Research Randomizer Version 4.0, Social Psychology Network, Wesleyan University, Middletown, CT, USA, https://www.randomizer.org/, accessed on 11 October 2023) in a 1:1 allocation. The order of the specific exercise tests was “pseudorandom” in that it was dictated through a combination of research participants’ availability for testing and the availability of appropriately skilled/qualified research personnel. considerations included the duration of the specific tests (i.e., the laboratory visits for vo_2peak_ and lactate threshold tests were of longer duration than the visits for time trials) and the need for a phlebotomist (i.e., only required for lactate threshold tests).

### 2.4. Screening

Potential research participants reported to the laboratory for an initial screening visit that consisted of a medical history/screening questionnaire, assessment of body mass and height, and measurement of VO_2peak_. Height was measured using a stadiometer, and body mass was measured using a physician’s scale. These data were used to calculate body mass index. VO_2peak_ was assessed during incremental cycle ergometer exercise (20–35 W/min) to voluntary fatigue using an electromagnetically braked ergometer (Corvial Cpet, Lode BV, Groningen, The Netherlands) and indirect calorimetry (ParvoMedics TrueOne 2400; Salt Lake City, UT, USA), as previously described [2,21,22]. 

### 2.5. Habituation

To habituate participants to the exercise protocols and avoid the confounding influence of a potential learning effect, two familiarization sessions were completed. These sessions were almost identical to the data collection visits described below; blood collection and placebo/lactate ingestion were not included in the habituation sessions.

### 2.6. VO_2peak_ and Ventilatory and Lactate Thresholds

VO_2peak_ was assessed during incremental cycle ergometer exercise (20–35 W/min) to voluntary fatigue using an electromagnetically braked ergometer and indirect calorimetry. Exhaled gases were analyzed for volume and concentration of oxygen and carbon dioxide. VO_2peak_ was recorded as the greatest value for VO_2_ averaged over 30 s. Based on previous studies in our lab [23], the coefficient of variation for the measurement of VO_2peak_ is 3.1%. The ventilatory threshold was determined using established procedures [24]. Every 60 s, arterialized venous blood (~2–3 mL) was sampled from a venous catheter placed in a dorsal hand vein; the hand and forearm were wrapped in a heated blanket [25]. Blood was immediately transferred to pre chilled tubes coated with sodium fluoride and potassium oxalate that were then returned to an ice slurry. Within 30 min of blood collection, lactate concentration was determined using an automated analyzer (YSI 2900, Xylem Inc.; White Plains, NY, USA). The work rate at the lactate threshold was identified as previously described [26]. In addition to gas exchange variables and blood lactate, heart rate (via short-range telemetry; Polar T31, Bethpage, New York, NY, USA) and ratings of perceived exertion (RPE; Borg scale) [27] were determined throughout the test. Once the participants had reached volitional fatigue, participants completed a verification phase [28]. The work rate was decreased (to ~0–50 watts at the request of the participants) to provide temporary (2 to 4 min) reprieve from strenuous exercise. The work rate was then rapidly increased (i.e., within seconds) to a value that would have been attained had the participant cycled for an additional 120 s beyond VO_2peak_ (e.g., if the ramp function was 25 W/min and a participant reached fatigue at 325 W, the verification work rate was increased to 375 W). Participants exercised at this higher work rate until volitional fatigue, at which point VO_2_ and blood lactate concentration were determined. 

### 2.7. Twenty-Minute Exercise Trial 

Participants completed a test very similar to an FTP^20^ [29], using an air-braked cycle ergometer (Concept2 BikeErg, Concept2 Inc., Morristown, VT, USA), as previously described [30]. This test involved the determination of the maximal mean power that could be sustained over 20 min. The FTP^20^ is able to predict cycling performance and is commonly used in laboratories and by cyclists of varied abilities (recreational to professional) to assess performance and training status [29]. RPE and heart rate were recorded at 5, 10, 15, and 20 min. To facilitate a maximal effort and to remove any potential ventilatory burden, expired gases were not collected during this test. Based on previous studies in our lab [30], the coefficient of variation for the FTP^20^ is 3.0%. 

### 2.8. Lactate Supplement

Both the placebo and the commercially available lactate supplement (Sportlegs, Sport Specifics, Inc., Longmont, CO, USA) were provided in capsules by the study sponsor (Sport Specifics, Inc., Loveland, CO, USA). To facilitate double blinding of all researchers and participants, capsules were delivered in containers labeled “A” and “B”, and the key was provided in a sealed envelope that was stored in a secure location by a colleague not affiliated with the project. Certificates of analysis were provided by a third party (New Generation Wellness, Inc., Colorado Springs, CO, USA). The ingredients of the lactate supplement were calcium lactate, magnesium lactate, and vitamin D3 (cholecalciferol); the total lactate per capsule was 372 mg. Within the USA, these ingredients are generally regarded as safe (i.e., satisfy the criteria for GRAS status) by the USA Food and Drug Administration. The placebo was organic rice starch. Visually, the placebo and lactate supplement were indistinguishable. To maintain ecological validity, individual dosing was as per the manufacturers’ guidelines: one capsule per 50 lbs (22.7 kg) body mass, rounded up. For example, a participant with a body mass of 120 lbs received three capsules; a participant with a body mass of 185 lbs received four capsules, etc. 

### 2.9. Statistics

This was a pilot study thus sample size was not predetermined. Sequence and period effects specific to our crossover design were calculated following published guidelines [31]. R Studio (version 2023.03.0 + 386) was used to run linear mixed models to compare variables of interest with the lme4 and lmerTest packages [32,33]. “Subjects” was always considered a random effect, to account for repeated measures. The effect size metric used for the linear mixed models was conditional R^2^. For single-factor tests (e.g., VO_2peak_), Yuen’s test was used. For two-factor tests (e.g., heart rate through the 20-min exercise trial), both condition and time were considered fixed effects. Significant main effects were further investigated via Tukey’s Honestly Significant Difference tests using the emmeans package. Following an estimation statistics approach [34], the effect size as Hedges’ g and the corresponding 95% confidence intervals (CI) were calculated. Relations of interest were explored using Pearson correlation. All data are presented as mean ± standard deviation unless otherwise specified. Data were considered statistically significant when *p* < 0.05. Estimation plots were created using GraphPad Prism 10 (GraphPad Software, Inc., Boston, MA, USA). All other figures were created using SigmaPlot (Grafiti LLC., Palo Alto, CA, USA).

## 3. Results

**Figure 1:**
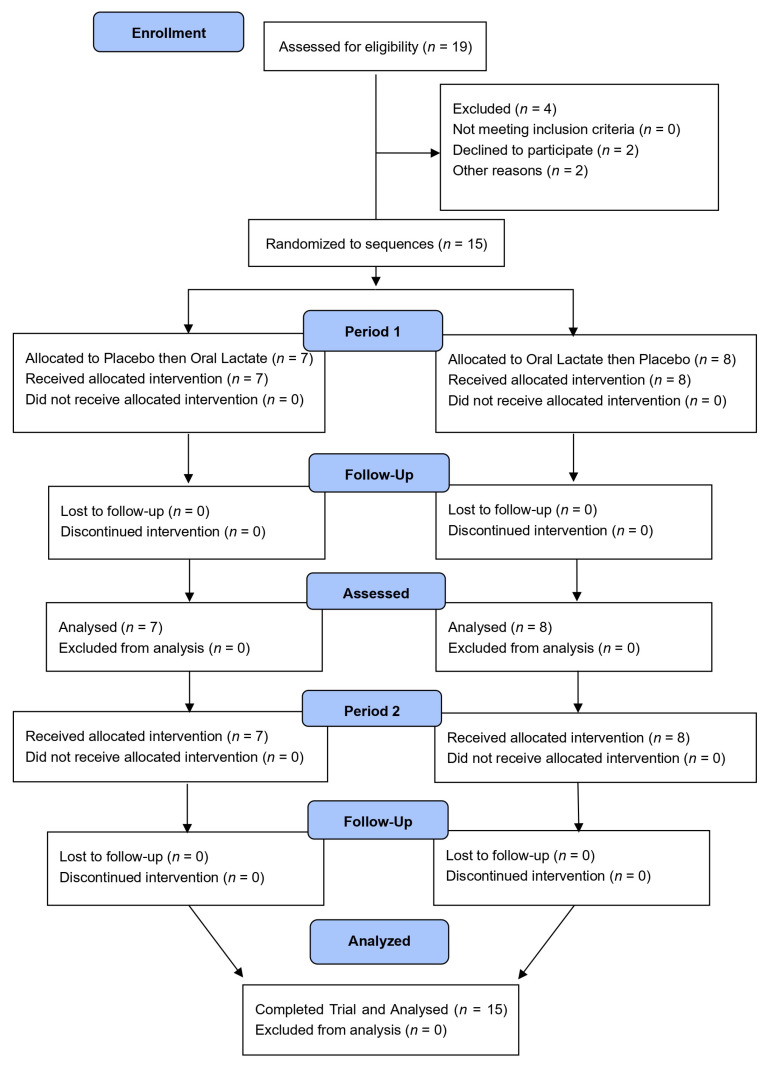
Consolidated standards of reporting trials (CONSORT) flow diagram for incremental exercise test.

**Figure 2:**
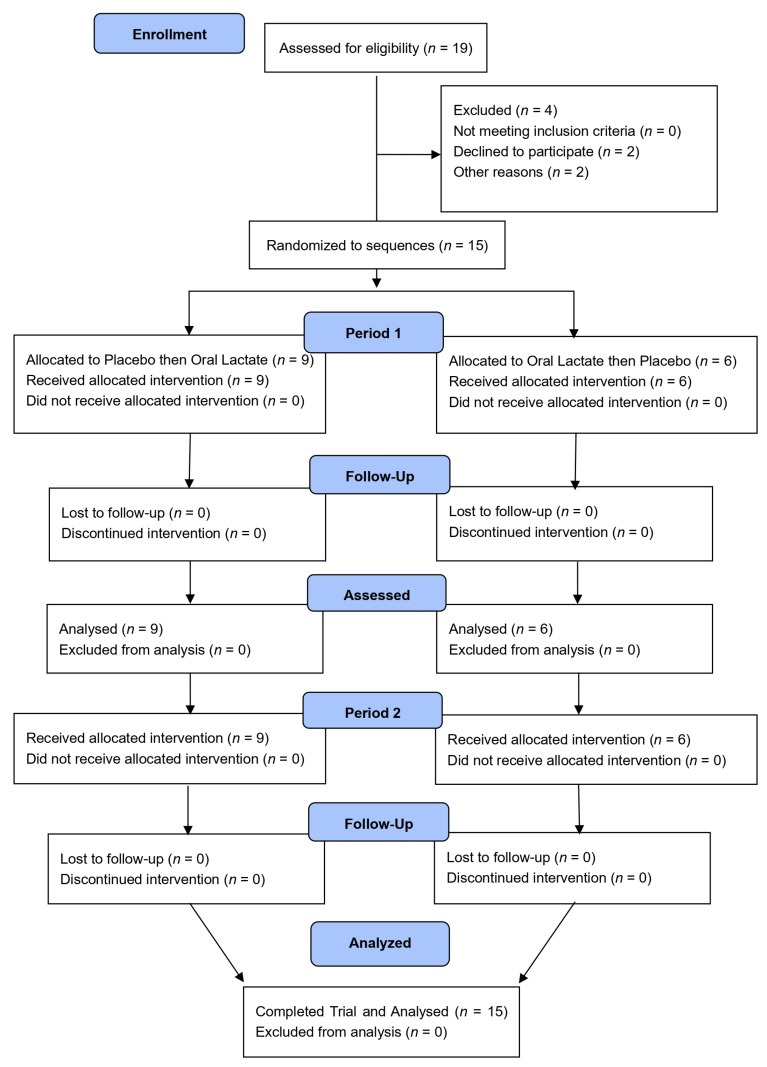
Consolidated standards of reporting trials (CONSORT) flow diagram for FTP^20^.

Participant recruitment and data collection and analysis took place between October 2023 and June 2024. The participant flow through the protocol is presented in Figure 1 (Incremental Exercise test) and Figure 2 (Time Trial). Two flow diagrams are necessary as the placebo and oral lactate assignments were different between tests. Nineteen healthy young regular exercisers were enrolled, of which four ended their participation prematurely: two participants were unable to tolerate blood sampling, and two participants were lost to follow-up (unresponsive to scheduling emails and texts). The remaining fifteen completed the study without incident or adverse event. Study participants comprised nine males and six females (age: 24 ± 3 years; body mass: 75.5 ± 12.7 kg (~166 ± 28 lbs); body mass index: 24.8 ± 2.3 kg/m^2^ (mean ± standard deviation)). The ingested dose of lactate supplement was 19 ± 1 mg/kg. Based on published guidelines recommended for classifying research participants [35,36], the study cohort comprised four males and one female considered untrained (Performance Level 1), three males and four females considered recreationally trained or active (Performance Level 2), and two males and one female considered trained (Performance Level 3). None of the participants were considered well-trained or professional (Performance Levels 4 and 5, respectively).

### 3.1. Incremental Exercise Test

**Figure 3:**
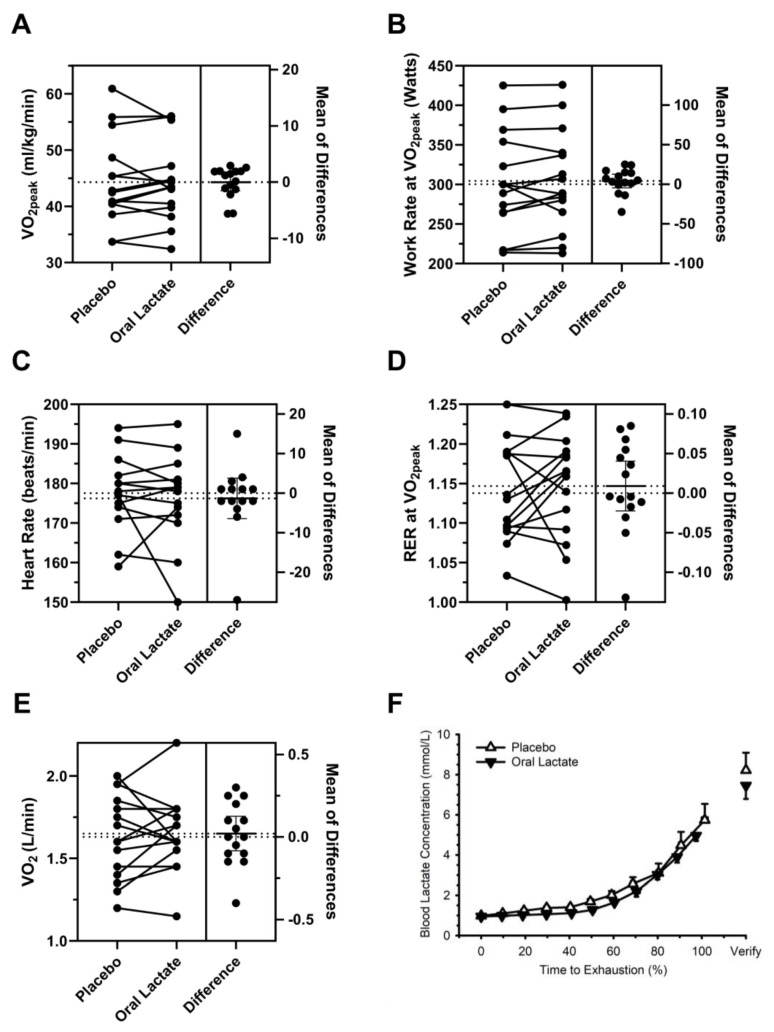
Oral lactate had no influence on the physiological responses to ramp exercise. Panels (**A**–**E**): The paired mean difference between placebo and oral lactate as illustrated using estimation plots. Both conditions are plotted on the left axes as a slopegraph: each paired set of observations is connected by a line. The paired mean difference is plotted on a floating axis on the right. Symbols represent individual mean differences, the horizontal line at zero provides reference, the other shows the actual mean difference. The error bars around the heavy solid horizontal line are the 95% confidence intervals. Panel (**A**): Oral lactate had no influence on peak oxygen uptake (VO_2peak_). Panel (**B**): Oral lactate had no influence on work rate at VO_2peak_. Panel (**C**): Oral lactate had no influence on heart rate at VO_2peak_. Panel (**D**): Oral lactate had no influence on respiratory exchange ratio (RER) at VO_2peak_. Panel (**E**): Oral lactate had no influence on the VO_2_ at ventilatory threshold. Panel (**F**): Oral lactate had no influence on blood lactate concentration during incremental exercise. Panels (**A**–**E**) show individual data, discriminated by sex. Panel (**F**) shows mean and standard deviation of group data. Exercise time was normalized to % of time to exhaustion to promote visual clarity.

Individual and group data from the incremental exercise test are presented in Figure 3A–F. One participant was unable to complete the verification phase in the oral lactate condition due to nausea during/after the VO_2peak_ assessment; it was unclear if this nausea was due to exercise, oral lactate, or some combination of both. Data from this participant were included in all formal statistical comparisons except those pertaining to the verification phase. There were no sequence or period effects for any of the presented variables (all *p* > 0.12). Compared with placebo, the oral lactate supplement did not influence VO_2peak_ (Panel A: placebo 44.3 ± 7.8 vs. oral lactate 44.3 ± 7.1 mL/kg/min; *p* = 0.87, Hedges’ g: 0.00 (95% CI −0.21, 0.18)), work rate at VO_2peak_ (Panel B: placebo 300 ± 64 vs. oral lactate 304 ± 62 W; *p* = 0.86, Hedges’ g: 0.06 (95% CI −0.08, 0.19)), heart rate at VO_2peak_ (Panel C: placebo 176 ± 10 vs. oral lactate 176 ± 11 beats/min; *p* = 0.94, Hedges’ g: −0.15 (95% CI −0.65, 0.24)), respiratory exchange ratio at VO_2peak_ (Panel D: placebo 1.14 ± 0.06 vs. oral lactate 1.15 ± 0.08; *p* = 0.47, Hedges’ g: 0.14 (95% CI −0.36, 0.61)), VO_2_ at ventilatory threshold (Panel E: placebo 1.63 ± 0.25 vs. oral lactate 1.65 ± 0.23 L/min; *p* = 0.82, Hedges’ g: 0.08 (95% CI −0.37, 0.45)), or the blood lactate response during exercise (Panel F). Further inspection of blood lactate data revealed no difference between placebo and the oral lactate supplement on pre-exercise (post capsule ingestion) blood lactate concentration (placebo 0.99 ± 0.33 vs. oral lactate 0.95 ± 0.24 mmol/L; *p* = 0.98, Hedges’ g: −0.12 (95% CI −0.85, 0.70)), blood lactate concentration at the end of the verification phase (placebo 8.22 ± 2.90 vs. oral lactate 7.46 ± 2.11 mmol/L; *p* = 0.40, Hedges’ g: −0.21 (95% CI −0.70, 0.29)), and work rate at the lactate threshold (placebo 179 ± 69 vs. oral lactate 179 ± 59 Watts; *p* = 0.41, Hedges’ g: 0.04 (95% CI −0.461, 0.744)). In addition, oral lactate supplementation had no effect on time to fatigue during the ramp test (placebo 624 ± 59 vs. oral lactate 635 ± 60 s; *p* = 0.85, Hedges’ g: 0.19 (95% CI −0.148, 0.444)), or during the verification phase (placebo 40 ± 20 vs. oral lactate 47 ± 32 s; *p* = 0.75, Hedges’ g: 0.24 (95% CI −0.197, 0.871)). VO_2_ at the end of the verification phase was slightly lower than VO_2peak_ for both treatments (placebo: 44.3 ± 7.8 vs. 39.6 ± 6.3 and oral lactate 44.3 ± 7.1 vs. 39.0 ± 7.7 mL/kg/min; *p* < 0.001); oral lactate supplementation did not influence this response (*p* = 0.69). Visual inspection of Panels C and D (heart rate and RER at VO_2peak_) suggested one participant had an unusual/exaggerated response to oral lactate (i.e., appreciably lower heart rate and RER). This participant’s data were not considered as outliers, nor were there any reported or observed peculiarities that would cause us to consider excluding this participant. However, for sake of clarity, we repeated the statistical analyses with these data excluded. While this resulted in minor changes in overall *p*-values, none of the conclusions were modified (i.e., comparisons that were previously interpreted as statistically significant/non-significant did not change).

### 3.2. Twenty-Minute Exercise Time Trial (FTP^20^)

**Figure 4:**
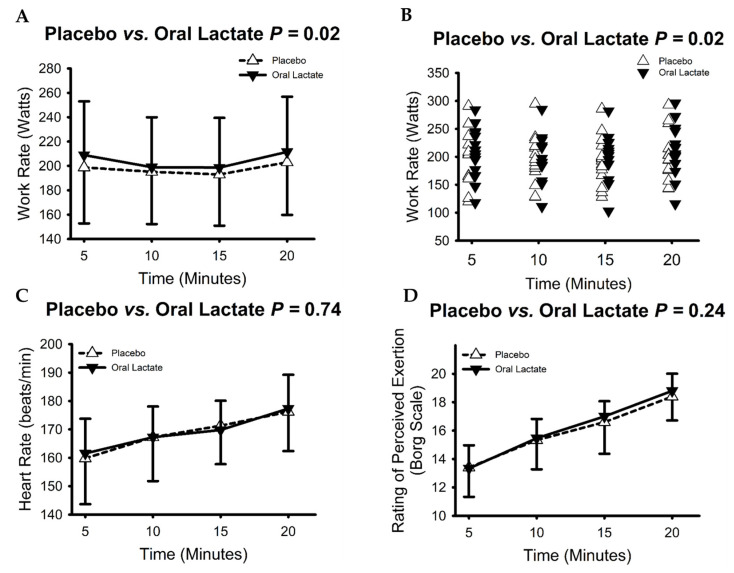
Oral lactate evoked a modest improvement in 20-min cycle ergometer time trial performance without influencing heart rate or ratings of perceived exertion (RPE). Panels (**A**,**B**) show group (**A**) and individual (**B**) data for work rates over the 20-min trial. Panel (**C**): Oral lactate had no influence on heart rate during the time trial. Panel (**D**): Oral lactate had no influence on RPE during the time trial. Panels (**A**,**C**,**D**) show mean and standard deviation of group data. Panel (**B**) shows individual responses; data have been offset from 5-min markers to promote visual clarity.

Group and individual data from the 20-min exercise time trial are presented in Figure 4A–D. There were no sequence or period effects for any of the presented variables (all *p* > 0.073). Compared with placebo, oral lactate supplementation improved mean work rate sustained through the 20-min exercise trial (placebo 197 ± 41 vs. oral lactate 204 ± 41 Watts; main effect of treatment *p* = 0.02, Conditional R^2^: 0.87; Panel A shows group data and Panel B individual data). Inspection of the individual data revealed that time trial performance was improved in eleven participants and decreased in the remaining four (3 males and one female). The mean change in performance with oral lactate was 7 ± 13 Watts (range: −18 to +26 Watts) corresponding to an overall 4% improvement. The magnitude of improvement in performance was not related to VO_2peak_, mean work rate, work rate at lactate threshold, or dose (mg/kg) of oral lactate (all *p* > 0.06). During the time trial, heart rate (Panel C-group data) increased over time (*p* < 0.001, Conditional R^2^: 0.86) and was not different between placebo and oral lactate supplementation (*p* = 0.74). Similarly, RPE (Panel D-group data) increased over time (*p* < 0.001, Conditional R^2^: 0.85) and was also not different between placebo and oral lactate supplementation (*p* = 0.24).

## 4. Discussion

The major findings of the current study were, compared with placebo, oral lactate supplementation did not influence any of the physiological responses during incremental exercise to fatigue, including VO_2peak_, VO_2_ at ventilatory threshold, work rate at lactate threshold, and work rate at VO_2peak_. However, during the 20-min time trial (FTP^20^), the sustained work rate was modestly (4%) increased with the oral lactate supplement, suggestive of a potential ergogenic effect.

Previous studies of oral lactate supplements in human exercise trials have yielded mixed results. Direct comparisons with the current data are problematic as dosing, the nature of the exercise, and the primary outcomes appear inconsistent across studies. For example, time to exhaustion during constant load (low-to-moderate intensity) exercise was unaffected by the addition of lactate to a carbohydrate sports drink [17,18]. Bicarbonate and pH were greater during three hours of constant load cycling following ingestion of a polylactate solution [19]. Time to exhaustion during short-duration, high-intensity treadmill exercise was barely extended (<2%) by high doses of lactate ingestion [6]. Neither 20- nor 40-km time trial performance was influenced by lactate ingestion [8,9]. Time to exhaustion during high-intensity cycle ergometer exercise was appreciably extended (17%) by high doses of oral lactate [5]. More recently, other commercially available supplements containing lactate have been shown to have unappreciable effects on skeletal muscle endurance during resistance exercise [37,38] but interpretation pertaining to lactate supplementation per se was complicated by the addition of other potentially active ingredients.

Major differences between the current and previous study [5] reporting a 17% extension of time to exhaustion pertain to dosing and exercise. In the previous study, 120 mg/kg of lactate was ingested; this dose was approximately six-fold greater than the dose used in the current study (19 ± 1 mg/kg). The rationale for our dose was based on a desire to maintain ecological validity by providing the dose recommended by the manufacturer and presumably used by the product consumers. In contrast, the dose used in the previous study was based on pilot dose-response investigations comparing circulating bicarbonate concentrations following ingestion of 20, 120, and 220 mg/kg of lactate. Thus, it appears plausible the differences between the magnitudes of relative improvements (4 vs. 17%) between the two studies could be attributed to vastly different dosing regimens. In support of this notion, two other studies using similar doses of the same commercially available lactate supplement used in the current study, showed no ergogenic benefit to 20- and 40-km time trial performance [8,9]. However, dosing may not be the most critical explanation as in a separate study, large doses of lactate (400 mg/kg sodium lactate) evoked only small improvements (~3 s) in time to exhaustion during treadmill sprinting [6]. An alternate explanation pertains to the selection of exercise testing protocols. The previous studies appear to be focused on the buffering abilities of lactate together with its properties as a versatile fuel source. To best exploit these characteristics, one might consider an exercise protocol that provided a challenge to acid-base balance regulation while also stressing the relatively limited stores of muscle glycogen. Our rationale for incorporating a 20-min, FTP^20^ style time trial was exercise of similar type and duration decreases skeletal muscle glycogen [39,40,41] and lowers pH [42,43]. The 20-min time trial is also a popular test frequently used by cyclists [29]. The study that has shown the greatest ergogenic effect of oral lactate used an exercise protocol comprising time to exhaustion during high-intensity exercise, preceded by multiple 60-s intervals of cycling at 100% of maximal power output [5]. We felt that the 20-min time trial was more reflective of the demands of competition than time to exhaustion, and evokes physiological challenges that, in theory, could be alleviated with lactate supplementation. Based on the current and previous studies of oral lactate supplementation [5,8,9], future investigators may wish to use shorter-duration, higher-intensity protocols. 

To the best of our knowledge, we are the first to demonstrate that low-dose lactate ingestion has no influence on the physiological responses to incremental exercise to voluntary fatigue. The absence of an effect on VO_2peak_ is not necessarily unsurprising as lactate per se is not usually considered a determinant of peak metabolic rate; rather, the delivery and utilization of oxygen drive this parameter. In contrast, and almost implied by definition, lactate availability should have a direct influence on the lactate threshold and yet our data revealed a negligible effect. There are several potential explanations that are not necessarily mutually exclusive. These include unchanged lactate availability on account of metabolism of the ingested lactate within the gut, and/or insignificant transport of ingested lactate into the circulation. Other studies have also reported no change in circulating lactate following lactate ingestion [5,6,8,9]. Additional proposed explanations pertained to the pharmacokinetics of ingested lactate (i.e., time to peak lactate following ingestion) or the possibility that the dose of ingested lactate relative to endogenous lactate production was trivial. Isotopic tracer studies would be required to definitively address these questions. 

Careful consideration of the study population is important for ergogenic aid studies. Interventions that benefit elite athletes may not always provide similar benefits to recreational exercisers, and vice versa. In the current study, although a history of regular exercise was the primary inclusion criteria, our study population comprised people considered to be untrained, recreationally trained or active, and trained [35,36]. We considered the influence of endurance training status on the ergogenic effect of oral lactate by exploring statistical relations between the magnitude of improvement in time trial performance, and VO_2peak_, lactate threshold, and baseline time trial performance; none of these relations were significant (all *p* > 0.06). These statistics might imply that training status and baseline athletic ability may not be determinants of the response to oral lactate supplementation.

Work rates during the 20-min time trial were increased by 4% (7 Watts). While this magnitude of improvement is small, it is worth considering that the difference between athletes who do and do not finish competitive events on the winners’ podium are often also very small, sometimes only seconds apart. Alternatively, the participants in the current study were mostly recreational exercisers, and therefore these small improvements with the oral lactate supplement may not be as relevant. Irrespective, the observation that work rate was slightly increased in the absence of any change in perceived exertion or heart rate may have greater direct implications for training than for competition. That is, if moderately greater training stimulation can be provided to skeletal muscle without the need for greater effort, this may eventually favorably modify performance during competition. Recent training studies in rodents coupled with long-term lactate administration provide support for this notion [10,11]. 

### Limitations 

There are several limitations in the current study that warrant further consideration. The primary limitation pertains to lactate dosing. As previously stated, we wished to use the dose recommended by the manufacturer to promote ecological validity and the overall applicability of our findings. Similar approaches in studies of the same lactate supplement have been followed [8,9]. Unfortunately, the guidelines provided by the manufacturer appear somewhat crude and potentially too small. The recommendation by the manufacturer to ingest one capsule per 50 lbs of body mass, rounded up to the next 50 lbs increment, likely contributed to variability between participants. That is, participants with body mass close to, but either side of 50 lbs thresholds may have received appreciably different doses. To illustrate, one participant weighed 149 lbs (67.7 kg) and received three capsules, but another participant weighed 153 lbs (69.5 kg) and received four capsules. When considered relative to body mass, these guidelines produced different doses (16.5 vs. 21.4 mg/kg). It is plausible that variability between participants with respect to lactate dosing contributed to the overall variability in the time-trial performance. However, this appears unlikely as there was no relation between lactate dose and the magnitude of improvement in time trial work rate with oral lactate. Instead, considering the larger doses used in other studies [5], it appears more likely that the dose delivered in the current study was insufficient to evoke a stronger ergogenic effect. Somewhat related, during participant recruitment, we did not consider recent changes in body mass as part of the exclusion criteria. This oversight could have contributed to increased inter-/intra-participant variability in dosage, especially in participants whose change in mass resulted in crossing a 50-pound threshold. Fortunately, no participants changed body mass during the period spanning data collection, thus intra-participant dosing was constant across trials. 

Other potential limitations include an absence of consideration for menstrual phase in the female participants, and standardization of pre-exercise nutrition in the total study population. Both menstrual phase [44] and pre-exercise nutrition [45] can influence athletic performance. While we acknowledge these limitations, we believe their influence on our data to be minimal on account of the randomization of treatments, thereby removing any systematic bias, and our surmise that inconsistencies in circulating sex hormones in the females collectively had less of an influence on the data than oral lactate ingestion.

Although the magnitude of improvement in time-trial performance with oral lactate was not related to VO_2peak_, mean work rate, work rate at lactate threshold, or dose (mg/kg) of oral lactate (all *p* > 0.06), given that *p*-values of bivariate correlations are influenced by sample size, it appears possible that in a larger cohort some of these variables may have been significantly associated with the magnitude of improvement. Thus, significant associations, if present, may be revealed in future studies employing larger population cohorts. 

The collection of blood for quantification of parameters other than lactate, such as pH and bicarbonate, may have provided useful insight into potential mechanisms contributing to the observed moderate ergogenic effect. Unfortunately, these analyses were not feasible on account of the available funds supporting the project.

Finally, additional considerations that may be relevant for future work but were beyond the scope of the current study include characterization of the gut microbiota and the potential influence of genetic polymorphisms encoding for monocarboxylate transporters (MCTs). With respect to the former, the gut microbiota is likely to play an important role in the metabolism of ingested lactate [46], and exercise training status is a known modifier of the gut microbiota [47,48]. It is plausible that differences may exist in the responses to oral lactate between adults who are recreationally trained compared with well-trained and professional athletes. As for the latter, MCTs are critical to the regulation of lactate and its transport between anatomical compartments. The function of MCTs is in part determined by genetics. Polymorphisms of MCTs influence athletic performance and responses to exercise, including lactate and ventilatory thresholds and substrate utilization [49,50,51]. In the context of the current study, responses to oral lactate may also be partially determined by specific gene variants of MCTs. This may be a useful consideration when contemplating variability between participants in future studies. 

## 5. Conclusions

Low dose oral lactate ingestion appears to have neither ergolytic nor ergogenic effects on the physiological responses to incremental exercise in healthy young adult habitual exercisers. However, the same oral lactate dose was able to evoke a modest ergogenic effect during a 20-min time trial. In the context of athletic performance and competition, sometimes a small advantage can be sufficient to achieve success. Given the heterogeneity and size of the study cohort, the relevance of this final statement should be interpreted with caution.

## Data Availability

The data presented in this study are available on request from the corresponding author.

## References

[1] Nalbandian M., Takeda M. (2016). Lactate as a Signaling Molecule That Regulates Exercise-Induced Adaptations. Biology.

[2] Abbotts K.S.S., Ewell T.R., Bomar M.C., Butterklee H.M., Bell C. (2023). Caffeine Augments the Lactate and Interleukin-6 Response to Moderate-Intensity Exercise. Med. Sci. Sport. Exerc..

[3] Hojman P., Brolin C., Norgaard-Christensen N., Dethlefsen C., Lauenborg B., Olsen C.K., Abom M.M., Krag T., Gehl J., Pedersen B.K. (2019). IL-6 release from muscles during exercise is stimulated by lactate-dependent protease activity. Am. J. Physiol. Endocrinol. Metab..

[4] Villarroya J., Campderros L., Ribas-Aulinas F., Carriere A., Casteilla L., Giralt M., Villarroya F. (2018). Lactate induces expression and secretion of fibroblast growth factor-21 by muscle cells. Endocrine.

[5] Morris D.M., Shafer R.S., Fairbrother K.R., Woodall M.W. (2011). Effects of lactate consumption on blood bicarbonate levels and performance during high-intensity exercise. Int. J. Sport. Nutr. Exerc. Metab..

[6] Van Montfoort M.C., Van Dieren L., Hopkins W.G., Shearman J.P. (2004). Effects of ingestion of bicarbonate, citrate, lactate, and chloride on sprint running. Med. Sci. Sport. Exerc..

[7] Brooks G.A. (2023). What the Lactate Shuttle Means for Sports Nutrition. Nutrients.

[8] Northgraves M.J., Peart D.J., Jordan C.A., Vince R.V. (2014). Effect of lactate supplementation and sodium bicarbonate on 40-km cycling time trial performance. J. Strength Cond. Res..

[9] Peveler W.W., Palmer T.G. (2012). Effect of magnesium lactate dihydrate and calcium lactate monohydrate on 20-km cycling time trial performance. J. Strength Cond. Res..

[10] Jang I., Kyun S., Hwang D., Kim T., Lim K., Park H.Y., Kim S.W., Kim J. (2024). Chronic Administration of Exogenous Lactate Increases Energy Expenditure during Exercise through Activation of Skeletal Muscle Energy Utilization Capacity in Mice. Metabolites.

[11] Takahashi K., Kitaoka Y., Yamamoto K., Matsunaga Y., Hatta H. (2020). Oral Lactate Administration Additively Enhances Endurance Training-Induced Increase in Cytochrome C Oxidase Activity in Mouse Soleus Muscle. Nutrients.

[12] Chiolero R., Mavrocordatos P., Burnier P., Cayeux M.C., Schindler C., Jequier E., Tappy L. (1993). Effects of infused sodium acetate, sodium lactate, and sodium beta-hydroxybutyrate on energy expenditure and substrate oxidation rates in lean humans. Am. J. Clin. Nutr..

[13] Bouzat P., Sala N., Suys T., Zerlauth J.B., Marques-Vidal P., Feihl F., Bloch J., Messerer M., Levivier M., Meuli R. (2014). Cerebral metabolic effects of exogenous lactate supplementation on the injured human brain. Intensive Care Med..

[14] Schiffer T., Schulte S., Sperlich B., Achtzehn S., Fricke H., Struder H.K. (2011). Lactate infusion at rest increases BDNF blood concentration in humans. Neurosci. Lett..

[15] Searle G.L., Feingold K.R., Hsu F.S., Clark O.H., Gertz E.W., Stanley W.C. (1989). Inhibition of endogenous lactate turnover with lactate infusion in humans. Metabolism.

[16] Haesler E., Schneiter P., Temler E., Jequier E., Tappy L. (1995). Effects of lactate infusion on hepatic gluconeogenesis and glycogenolysis. Clin. Physiol..

[17] Bryner R.W., Hornsby W.G., Chetlin R., Ullrich I.H., Yeater R.A. (1998). Effect of lactate consumption on exercise performance. J. Sport. Med. Phys. Fit..

[18] Swensen T., Crater G., Bassett D.R., Howley E.T. (1994). Adding polylactate to a glucose polymer solution does not improve endurance. Int. J. Sport. Med..

[19] Fahey T.D., Larsen J.D., Brooks G.A., Colvin W., Henderson S., Lary D. (1991). The effects of ingesting polylactate or glucose polymer drinks during prolonged exercise. Int. J. Sport. Nutr..

[20] Dwan K., Li T., Altman D.G., Elbourne D. (2019). CONSORT 2010 statement: Extension to randomised crossover trials. BMJ.

[21] Ewell T.R., Abbotts K.S.S., Williams N.N.B., Butterklee H.M., Bomar M.C., Harms K.J., Rebik J.D., Mast S.M., Akagi N., Dooley G.P. (2021). Pharmacokinetic Investigation of Commercially Available Edible Marijuana Products in Humans: Potential Influence of Body Composition and Influence on Glucose Control. Pharmaceuticals.

[22] Newman A.A., Grimm N.C., Wilburn J.R., Schoenberg H.M., Trikha S.R.J., Luckasen G.J., Biela L.M., Melby C.L., Bell C. (2018). Influence of Sodium Glucose Co-Transporter 2 Inhibition On The Physiological Adaptation to Endurance Exercise Training. J. Clin. Endocrinol. Metab..

[23] Richards J.C., Lonac M.C., Johnson T.K., Schweder M.M., Bell C. (2010). Epigallocatechin-3-gallate Increases Maximal Oxygen Uptake in Adult Humans. Med. Sci. Sport. Exerc..

[24] Beaver W.L., Wasserman K., Whipp B.J. (1986). A new method for detecting anaerobic threshold by gas exchange. J. Appl. Physiol..

[25] Forster H.V., Dempsey J.A., Thomson J., Vidruk E., DoPico G.A. (1972). Estimation of arterial PO2, PCO2, pH, and lactate from arterialized venous blood. J. Appl. Physiol..

[26] Wasserman K. (1986). The anaerobic threshold: Definition, physiological significance and identification. Adv. Cardiol..

[27] Borg G.A. (1982). Psychophysical bases of perceived exertion. Med. Sci. Sport. Exerc..

[28] Midgley A.W., Carroll S. (2009). Emergence of the verification phase procedure for confirming ‘true’ VO(2max). Scand. J. Med. Sci. Sport..

[29] Mackey J., Horner K. (2021). What is known about the FTP(20) test related to cycling? A scoping review. J. Sport. Sci..

[30] Ewell T.R., Bomar M.C., Abbotts K.S.S., Butterklee H.M., Dooley G.P., Bell C. (2022). Edible marijuana and cycle ergometer exercise. Front. Physiol..

[31] Lim C.Y., In J. (2021). Considerations for crossover design in clinical study. Korean J. Anesthesiol..

[32] Bates D., Mächler M., Bolker B., Walker S. (2015). Fitting Linear Mixed-Effects Models Using lme4. J. Stat. Softw..

[33] Kuznetsova A., Brockhoff P.B., Christensen R.H.B. (2017). lmerTest Package: Tests in Linear Mixed Effects Models. J. Stat. Softw..

[34] Ho J., Tumkaya T., Aryal S., Choi H., Claridge-Chang A. (2019). Moving beyond P values: Data analysis with estimation graphics. Nat. Methods.

[35] Decroix L., De Pauw K., Foster C., Meeusen R. (2016). Guidelines to Classify Female Subject Groups in Sport-Science Research. Int. J. Sport. Physiol. Perform..

[36] De Pauw K., Roelands B., Cheung S.S., de Geus B., Rietjens G., Meeusen R. (2013). Guidelines to classify subject groups in sport-science research. Int. J. Sport. Physiol. Perform..

[37] Xu J., Farney T.M., Nelson A.G. (2020). Muscle Sentry(R) has No Effect on Total Work Performed and Estimated MVO (2) after High Intensity Short Duration Resistance Training. Int. J. Exerc. Sci..

[38] Bartschi T.M., Sanders D.C., Farney T.M., Kokkonen J., Nelson A.G. (2017). A Pre-Exercise Dose of Muscle Sentry((R)) has no Effect on Performing Repeated Leg Press Sets to Failure. Int. J. Exerc. Sci..

[39] Dyck D.J., Peters S.J., Wendling P.S., Chesley A., Hultman E., Spriet L.L. (1996). Regulation of muscle glycogen phosphorylase activity during intense aerobic cycling with elevated FFA. Am. J. Physiol..

[40] McConell G.K., Lee-Young R.S., Chen Z.P., Stepto N.K., Huynh N.N., Stephens T.J., Canny B.J., Kemp B.E. (2005). Short-term exercise training in humans reduces AMPK signalling during prolonged exercise independent of muscle glycogen. J. Physiol..

[41] Gollnick P.D., Armstrong R.B., Saubert C.W.t., Sembrowich W.L., Shepherd R.E., Saltin B. (1973). Glycogen depletion patterns in human skeletal muscle fibers during prolonged work. Pflug. Arch. Eur. J. Physiol..

[42] Poffe C., Wyns F., Ramaekers M., Hespel P. (2021). Exogenous Ketosis Impairs 30-min Time-Trial Performance Independent of Bicarbonate Supplementation. Med. Sci. Sport. Exerc..

[43] Heck K.L., Potteiger J.A., Nau K.L., Schroeder J.M. (1998). Sodium bicarbonate ingestion does not attenuate the VO2 slow component during constant-load exercise. Int. J. Sport. Nutr..

[44] Oxfeldt M., Frederiksen L.K., Gunnarson T., Hansen M. (2024). Influence of menstrual cycle phase and oral contraceptive phase on exercise performance in endurance-trained females. J. Sport. Med. Phys. Fit..

[45] Clayton D.J., Barutcu A., Machin C., Stensel D.J., James L.J. (2015). Effect of Breakfast Omission on Energy Intake and Evening Exercise Performance. Med. Sci. Sport. Exerc..

[46] Scheiman J., Luber J.M., Chavkin T.A., MacDonald T., Tung A., Pham L.D., Wibowo M.C., Wurth R.C., Punthambaker S., Tierney B.T. (2019). Meta-omics analysis of elite athletes identifies a performance-enhancing microbe that functions via lactate metabolism. Nat. Med..

[47] Petri C., Mascherini G., Izzicupo P., Rosati D., Cerboneschi M., Smeazzetto S., Arrones L.S. (2024). Gut microbiota and physical activity level: Characterization from sedentary to soccer players. Biol. Sport..

[48] Chen Y., Yang K., Xu M., Zhang Y., Weng X., Luo J., Li Y., Mao Y.H. (2024). Dietary Patterns, Gut Microbiota and Sports Performance in Athletes: A Narrative Review. Nutrients.

[49] Gasser B., Dossegger A., Giraud M.N., Fluck M. (2024). T-Allele Carriers of Mono Carboxylate Transporter One Gene Polymorphism rs1049434 Demonstrate Altered Substrate Metabolization during Exhaustive Exercise. Genes.

[50] Chavez-Guevara I.A., Gonzalez-Rodriguez E., Moreno-Brito V., Perez-Leon J.A., Amaro-Gahete F.J., Trejo-Trejo M., Ramos-Jimenez A. (2024). The polymorphism T1470A of the SLC16A1 gene is associated with the lactate and ventilatory thresholds but not with fat oxidation capacity in young men. Eur. J. Appl. Physiol..

[51] Pasqualetti M., Onori M.E., Canu G., Moretti G., Minucci A., Baroni S., Mordente A., Urbani A., Galvani C. (2022). The Relationship between ACE, ACTN3 and MCT1 Genetic Polymorphisms and Athletic Performance in Elite Rugby Union Players: A Preliminary Study. Genes.

